# Genome-Wide Gene Amplification during Differentiation of Neural Progenitor Cells *In Vitro*

**DOI:** 10.1371/journal.pone.0037422

**Published:** 2012-05-14

**Authors:** Ulrike Fischer, Andreas Keller, Meike Voss, Christina Backes, Cornelius Welter, Eckart Meese

**Affiliations:** Department of Human Genetics, Saarland University, Homburg/Saar, Germany; City of Hope National Medical Center and Beckman Research Institute, United States of America

## Abstract

DNA sequence amplification is a phenomenon that occurs predictably at defined stages during normal development in some organisms. Developmental gene amplification was first described in amphibians during gametogenesis and has not yet been described in humans. To date gene amplification in humans is a hallmark of many tumors. We used array-CGH (comparative genomic hybridization) and FISH (fluorescence in situ hybridization) to discover gene amplifications during *in vitro* differentiation of human neural progenitor cells. Here we report a complex gene amplification pattern two and five days after induction of differentiation of human neural progenitor cells. We identified several amplified genes in neural progenitor cells that are known to be amplified in malignant tumors. There is also a striking overlap of amplified chromosomal regions between differentiating neural progenitor cells and malignant tumor cells derived from astrocytes. Gene amplifications in normal human cells as physiological process has not been reported yet and may bear resemblance to developmental gene amplifications in amphibians and insects.

## Introduction

DNA sequence amplification describes any event that increases the copy number of a gene per haploid genome above the number that is characteristic for that organism [1]. Gene amplification has been reported for defined stages during normal development of *Xenopus laevis*, *Drosophila melanogaster*, *Sciara coprophila*, and *Tetrahymena thermophila* [1]–[4]. The ability of a cell to amplify genes represents an alternative to the problem of how best to meet a short and sharp, but heavy demand for a stage-specific protein [1]. This amplification process is spatially and temporally restricted on specific DNA regions and narrow windows of developmental time [4]. In humans, gene amplification has only been found in multidrug resistant cells and in cancer cells. The only circumstantial evidence for gene amplification in normal mammalian cells stems from two studies on mouse embryo cells with double minute chromosomes (DMs), that are cytogenetic manifestations of gene amplification. One study described DMs in cell lines derived from mouse fetus [5]. The other study described DMs in 1% of serum free mouse embryo (SFME) cells and an elevated frequency of DMs in cells grown in medium containing fetal calf serum (FCS) [6]. SFME cells were distributed from the American Type Culture Collection (ATCC) as neural stem cell line. From today's view, these SFME cells were neural progenitor cells that are capable of differentiating into astrocytes when grown in the presence of growth factor TGF-ß or fetal calf serum (FCS). This hint for gene amplification as physiological process in mammalian cells, specifically progenitor cells, prompted us to study gene amplification in normal human neural progenitor cells (NHNP). These cells grow as spheres and express β-III Tubulin (neuronal lineage) and GFAP (astrocyte lineage) upon differentiation. Recent studies on various human embryonic stem cells revealed genetic changes during prolonged culture [7]. Whole-genome genotyping analysis of NHNP cells created from NHNP primary cells at passage 20 were, however, still considered “normal” with a low number of CNVs (copy number variations)>200 kb (0–2 per line) [8], [9]. Notably, NHNP cells used in this study were primary cells in passage 1 [8].

The results of our genome-wide gene amplification analysis during *in vitro* differentiation of normal human progenitor cells revealed a complex amplification pattern after two and five days of differentiation. Representative examples of amplified chromosomal regions were confirmed by fluorescence in situ hybridizations. We further characterized those cells with amplifications using immunofluorescence staining. We found a strong overlap of amplified genes in neural progenitor cells undergoing differentiation and amplified genes in malignant tumors derived from astrocytes.

## Results

### Identification of gene amplifications using array-CGH analysis

**Figure 1:**
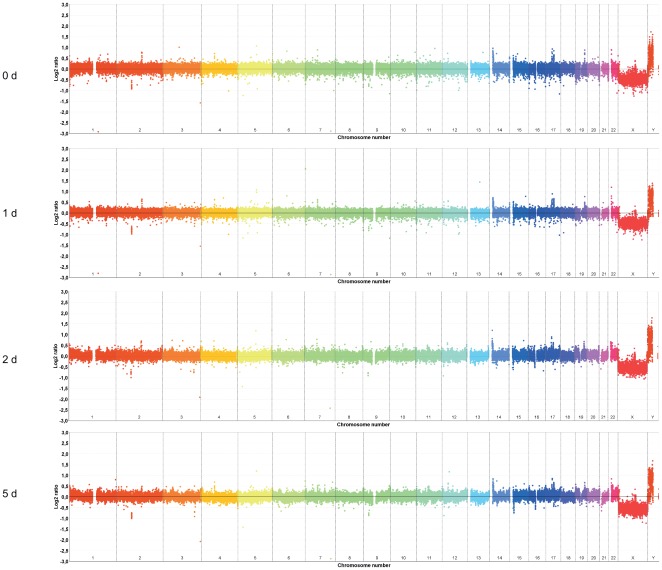
Whole chromosome plots. A genome-wide view of the 10× window-averaged data at 25 kb resolution is displayed for NHNP cells at day 0, day 1, day 2 and day 5 during differentiation.

Differentiation of NHNP cells was induced by withdrawal of EGF and bFGF and supplementation of brain derived neurotrophic factor (BDNF). The expression of Tubulin beta-3 chain and GFAP was analyzed by immunofluorescence after 24 h following differentiation induction. For amplification analysis total DNA was isolated from undifferentiated NHNP sphere cells and from NHNP cells differentiated for 24 h, 2 d and 5 d respectively and analyzed on NimbleGen 720K human whole genome tiling arrays. Signal intensity data were extracted from scanned images of each array using Roche NimbleGen NimbleScan v2.6 software. After spatial correction, the Cy3 and Cy5 signal intensities were normalized using qspline normalization. Following normalization a 10× window–averaging step is applied. Window-averaging reduces the size of the data and reduces the noise in the data. For amplification detection we used the dynamic segMNT algorithm that identifies segments by minimizing the squared error relative to the segment means. To detect representative alterations and to minimize the identification of random alterations, we extracted segments with segment means greater 0.1 threshold and a size greater than 250 kb. Chromosomal regions that revealed copy number gains and match CNVs (copy number variations) present in the Database of Genomic Variants available at UCSC Genome Browser were excluded from further analysis. While we did not detect amplified regions in NHNP cells at zero time and 24 h after differentiation, we found numerous chromosomal regions with copy number gains in NHNP cells after 2 d and 5 d of differentiation. In total we found 66 amplified chromosome regions after 2 d of differentiation and 93 amplified chromosome regions after 5 d of differentiation (Table 1). We also detected 9 deleted chromosome regions after 2 d of differentiation and 30 deleted chromosome regions after 5 d of differentiation. Whole genome profiles were presented in Figure 1.

**Table 1:** Overview on amplified chromosomal regions.

Amplified chromosomal regions after 2 d of differentiation					Amplified chromosomal regions after 5 d of differentiation					Genes
Chromo-some	start	end	log_2_ ratio	size (Mb)	Chromo-some	start	end	log_2_ ratio	size (Mb)	
chr1	362499	3837499	0,194	3,48	chr1	362499	3837499	0,175	3,48	
chr1	5612499	12612499	0,115	7,00	chr1	5662499	12187499	0,128	6,53	*PHF13*
chr1	151587499	155337499	0,125	3,75	**chr1**	**14362499**	**46587499**	**0,103**	**32,23**	
					chr1	151712499	155462499	0,161	3,75	***HDGF***
					**chr1**	**200612499**	**204962499**	**0,136**	**4,35**	***SOX13*** *,MDM4*
chr2	19937499	20737499	0,126	0,80	chr2	8462499	12112499	0,117	3,65	*ID2*
chr2	220037499	220312499	0,153	0,28	chr2	23412499	25337499	0,114	1,93	
					chr2	46937499	47712499	0,135	0,78	
					chr2	72912499	73337499	0,150	0,43	
					chr2	85237499	86737499	0,136	1,50	
					chr2	216662499	217312499	0,132	0,65	
chr3	46637499	50112499	0,117	3,48	chr3	8137499	16762499	0,104	8,63	
chr3	50137499	50687499	0,239	0,55	chr3	48212499	50712499	0,168	2,50	
chr3	51912499	52862499	0,164	0,95	chr3	51962499	53312499	0,163	1,35	
chr3	185087499	186037499	0,122	0,95	chr3	128537499	131287499	0,110	2,75	
chr3	194887499	196812499	0,104	1,93	chr3	184787499	186012499	0,131	1,23	
					chr3	194962499	199378356	0,105	4,42	
chr4	37499	9362499	0,103	9,33	chr4	637499	3737499	0,153	3,10	
					chr4	5812499	8537499	0,140	2,73	
chr5	87499	1912499	0,120	1,83	chr5	87499	1912499	0,103	1,83	*TERT*
chr5	149512499	150037499	0,149	0,53	chr5	10187499	10812499	0,121	0,63	
					chr5	137487499	142112499	0,131	4,63	
					chr5	148562499	150537499	0,120	1,98	*CAMK2A*
chr6	111987499	112287499	0,150	0,30	chr6	2662499	4112499	0,139	1,45	
					chr6	40362499	44862499	0,100	4,50	
**chr7**	**137499**	**2987499**	**0,140**	**2,85**						
chr7	43962499	45087499	0,127	1,13						
chr7	72012499	75887499	0,113	3,88						
**chr7**	**141837499**	**142212499**	**0,186**	**0,38**						
**chr7**	**150237499**	**150737499**	**0,166**	**0,50**						
chr8	27162499	27587499	0,127	0,43	chr8	37237499	38987499	0,102	1,75	
chr8	142112499	142612499	0,124	0,50	chr8	41562499	42312499	0,121	0,75	
chr8	143087499	146257115	0,120	3,17	chr8	61687499	62237499	0,148	0,55	
					chr8	123612499	124637499	0,103	1,03	
					chr8	140612499	146257115	0,127	5,64	
chr9	115812499	116412499	0,130	0,60	chr9	115787499	116412499	0,110	0,63	
chr9	122237499	140235768	0,104	18,00	chr9	122112499	124162499	0,110	2,05	
					chr9	128112499	140162499	0,149	12,05	
chr10	72962499	73787499	0,157	0,83	chr10	24837499	25287499	0,117	0,45	
chr10	79237499	81362499	0,125	2,13	chr10	78837499	81037499	0,146	2,20	
chr10	134437499	135087499	0,135	0,65	chr10	87812499	88812499	0,106	1,00	
					chr10	101912499	106112499	0,110	4,20	
**chr11**	**62499**	**4162499**	**0,112**	**4,10**	**chr11**	**62499**	**4162499**	**0,106**	**4,10**	
chr11	60737499	61387499	0,206	0,65	**chr11**	**17162499**	**17612499**	**0,142**	**0,45**	
					chr11	47112499	47587499	0,191	0,48	
					chr11	60187499	60712499	0,109	0,53	
					chr11	60987499	61537499	0,200	0,55	
					chr11	61912499	62412499	0,205	0,50	
					chr11	63362499	63812499	0,258	0,45	
					chr11	63837499	64112499	0,140	0,28	
					chr11	64412499	64887499	0,145	0,48	
					chr11	65312499	66587499	0,174	1,28	
chr12	6237499	7212499	0,136	0,98	chr12	6262499	7212499	0,174	0,95	
chr12	123362499	123687499	0,213	0,33	chr12	47312499	53112499	0,112	5,80	*HOXC6*
chr12	123712499	124462499	0,110	0,75	**chr12**	**54337499**	**56762499**	**0,147**	**2,43**	***CDK4, CYP27B1, KUB3***
					chr12	118937499	124487499	0,137	5,55	*SCARB1, * ***DIABLO***
					chr13	23412499	23812499	0,135	0,40	
					chr13	24362499	24687499	0,108	0,33	
chr14	22062499	24037499	0,117	1,98	chr14	22062499	24137499	0,138	2,08	
chr14	76412499	77062499	0,145	0,65						
chr15	18862499	21212499	0,133	2,35	chr15	18537499	20062499	0,139	1,53	
chr15	28237499	28662499	0,186	0,43	chr15	28237499	28637499	0,106	0,40	
chr15	29212499	29537499	0,148	0,33	chr15	29187499	29462499	0,128	0,28	
chr15	30237499	30612499	0,126	0,38	chr15	38062499	38537499	0,168	0,48	
chr15	72112499	73037499	0,160	0,93	chr15	49987499	50362499	0,105	0,38	
chr15	75687499	76137499	0,202	0,45	chr15	71787499	73387499	0,173	1,60	
chr15	80387499	80862499	0,186	0,48	chr15	75562499	76162499	0,170	0,60	
chr16	12499	1487499	0,221	1,48	chr16	87499	1362499	0,190	1,28	
chr16	1512499	5012499	0,119	3,50	chr16	1512499	4937499	0,142	3,43	*NUDT16L1*
chr16	29487499	31512499	0,151	2,03	chr16	29487499	31487499	0,138	2,00	
chr16	82487499	83737499	0,106	1,25	chr16	55437499	56687499	0,148	1,25	
chr16	83762499	84337499	0,222	0,58	chr16	64662499	69387499	0,121	4,73	
chr16	86587499	88707518	0,181	2,12	chr16	73762499	74137499	0,151	0,38	
					chr16	79962499	80287499	0,161	0,33	
					chr16	82487499	83712499	0,127	1,23	
					chr16	83737499	84412499	0,250	0,68	***GINS2***
					chr16	85862499	88707518	0,162	2,85	
chr17	12499	2937499	0,124	2,93	chr17	12499	2837499	0,136	2,83	
chr17	6862499	8337499	0,139	1,48	chr17	7037499	8162499	0,202	1,13	***TP53***
chr17	16262499	18037499	0,144	1,78	chr17	16037499	19787499	0,138	3,75	
chr17	22587499	24437499	0,101	1,85	chr17	23787499	24537499	0,182	0,75	
chr17	33687499	36012499	0,119	2,33	chr17	31812499	41512499	0,126	9,70	***C1QL1***
chr17	36812499	40937499	0,122	4,13	chr17	42137499	46812499	0,118	4,68	*ABCC3*
chr17	41037499	41537499	0,104	0,50	chr17	62137499	62462499	0,179	0,33	
chr17	46087499	46687499	0,113	0,60	chr17	67562499	78637061	0,151	11,07	
chr17	67587499	76512499	0,126	8,93						
chr17	76537499	76937499	0,291	0,40						
chr17	76962499	78637061	0,149	1,67						
chr18	54687499	54962499	0,126	0,28	chr18	33037499	33462499	0,103	0,43	
					**chr18**	**75287499**	**76107311**	**0,104**	**0,82**	
chr19	212499	8487499	0,145	8,28	chr19	37499	8612499	0,172	8,58	
chr19	9737499	19837499	0,131	10,10	chr19	9787499	14712499	0,153	4,93	
**chr19**	**49912499**	**50537499**	**0,199**	**0,63**	chr19	15062499	20137499	0,153	5,08	
					**chr19**	**37762499**	**47887499**	**0,101**	**10,13**	
					**chr19**	**50012499**	**56212499**	**0,138**	**6,20**	*FUT1*
**chr20**	**59037499**	**62387499**	**0,162**	**3,35**	**chr20**	**20137499**	**20412499**	**0,142**	**0,28**	
					**chr20**	**24887499**	**25687499**	**0,110**	**0,80**	
					**chr20**	**29462499**	**36962499**	**0,115**	**7,50**	
					**chr20**	**59137499**	**62387499**	**0,161**	**3,25**	
chr21	42387499	43537499	0,139	1,15	chr21	41887499	44337499	0,112	2,45	
chr22	18537499	18862499	0,357	0,33	**chr22**	**14862499**	**30862499**	**0,117**	**16,00**	*RANBP1*
chr22	33737499	49559126	0,108	15,82	chr22	33987499	49559126	0,127	15,57	

Chromosome regions that overlap with gained chromosome regions of TCGA glioblastoma samples are indicated in bold. Likewise, genes that were used for FISH analysis are indicated in bold. Examples of glioblastoma-amplified genes were included for chromosomal regions amplified after 5 d of differentiation. Start and end point were according to NCBI36/HG18.

### Confirmation of gene amplification of selected loci

**Figure 2:**
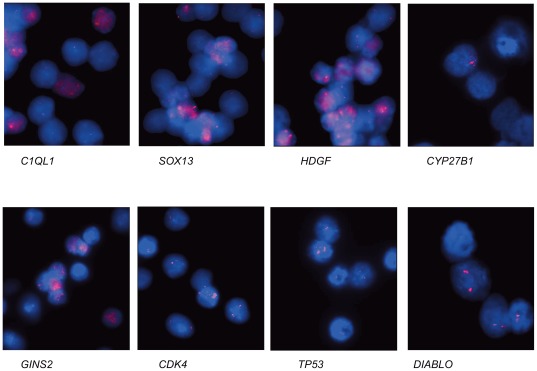
FISH analysis of amplified loci. For each FISH analysis, a BAC or cosmid clone containing the indicated gene was Cy3-labelled (pink) and hybridized against fixed NHNP cells that were differentiated for either 2, 5 or 7 days. Amplifications are shown for *C1QL1* RP11-113A24 (5 days), *SOX13* RP11876H8 (5 days), *HDGF* RP11-66D17 (5 days), *CYP27B1* cosmid (2 days), *GINS2* RP11-118F19 (2 days), *CDK4* RP11-571M6 (2 days), *TP53* RP11-1081A10 (7days), *DIABLO* RP11-568C23 (7 days). Nuclei were counterstained with DAPI. Size calibration bar = 5 µm. Notably, the degree of amplification various within each analysis due to the high heterogeneity of the amplifications in each cell population.

Using array-CGH we identified amplified chromosome regions in a mixed population of cells during *in vitro* differentiation. To validate these results we used fluorescence in situ hybridization (FISH) on loci with log_2_ ratios ranging from 0.126 to 0.250. We analyzed one gene for each locus including 1q23.1 at 154.98 Mb (*HDGF*), 1q32.1 at 202.35 Mb (*SOX13*), 12q14.1 at 56.43 Mb (*CDK4*), at 56.44 (*CYP27B1*) and at 56.63 Mb (*XRCC6BP1/KUB3*), 12q24.31 at 121.26 MB (*DIABLO*), 16q24.1 at 84.27 Mb (*GINS2*), 17p13.1 at 7.52 Mb (*TP53*) and 17q21.31 at 40.4 Mb (*C1QL1*). FISH analysis confirmed the amplifications for all loci (Figure 2). Next we determined the amplification frequency analyzing 150 nuclei per locus. We found an average amplification frequency for *GINS2* and *CDK4* of 5% after 2 d of differentiation, an amplification frequency for *CYP27B1* of 3% after 2 d of differentiation, an amplification frequency for *SOX13*, *C1QL1* and *HDGF* of 10% after 5 d of differentiation and an amplification frequency for *TP53* and *DIABLO* of 5% after 7 d of differentiation. Both the copy number variation in cells with gene amplification, and the absence of gene amplifications in many cells, account for the low increase in log_2_ ratio in array-CGH analysis.

**Figure 3:**
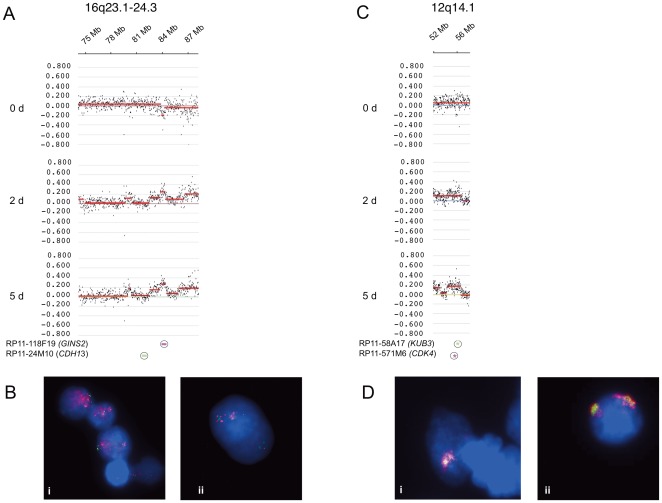
Detailed gene amplification analysis on human chromosome 16 and 12. Representative sections of log_2_ ratio profiles for undifferentiated (0 d) NHNP cells and cells that were differentiated for 2 and 5 days. Base count is given on the x-axis and log_2_ ratio on the y-axis for chromosome 16q24.1 (A) and 12q14.1 (C). Chromosomal localization of BAC probes used for FISH were indicated at the bottom of figures C and D. A *GINS2* specific BAC probe that was labeled in pink and a *CDH13* specific BAC probe that was labeled in green were hybridized simultaneously against fixed NHNP cells that were differentiated for 2 days. *GINS2* amplification is indicated as pink speckled fluorescence signals whereas the neighboring *CDH13* gene shows only single copy fluorescence signals (Bi). Neighboring cells with and without *GINS2* amplification are shown in Figure Bii. A *CDK4* specific BAC probe that was labeled in pink and a *XRCC6BP1/KUB3* specific BAC that was labeled in green were hybridized simultaneously against NHNP cells that were differentiated for 2 days. *CDK4* and *KUB3* amplifications were detectable as cluster of pink and green speckled fluorescence signals. *CDK4* specific signals spread over a more extended area than the *KUB3* specific signals (D). Nuclei were counterstained with DAPI (B). Size calibration bar = 5 µm.

As further validation step we compared the amplification event between genes from two neighboring chromosome regions. Within chromosome region 16q24.1 the log_2_ ratio values revealed an increase of genomic sequences at 83.7–84.4 Mb. In the same chromosome region, the log_2_ ratio values indicated a normal copy number at 82 Mb (Figure 3A). FISH experiments revealed amplification of the *GINS2* gene that maps at 84.27 Mb, but no amplification for *CDH13* at 82 MB (Figure 3Bi). FISH analysis also provided evidence for a large heterogeneity of amplifications in neighboring cells. Figure 3Bii shows a nucleus with amplified *GINS2* fluorescence signals next to a nucleus with only 3 *GINS2* specific fluorescence signals.

For further validation we analyzed chromosome region 12q14.1. For this region the log_2_ ratio values indicated an increase of genomic sequences at 54–56.7 Mb (Figure 3C). FISH confirmed not only the amplification of the *CDK4* gene at 56.43 Mb and the adjacent *XRCC6BP1/KUB3* gene at 56.63 Mb (Figure 3 Di) but also indicated the difference in the localization of the amplified sequences between both genes. While the *CDK4* specific fluorescence signals were widely spread, the *XRCC6BP1/KUB3* signals were more focused. (Figure 3 D ii).

### Characterization of cells with gene amplifications

We asked whether the identified amplification pattern was different between cells that were still part of the sphere and cells that migrated out of the sphere during the differentiation process. By using simultaneous FISH and immunofluorescence (IF) we analyzed the amplification status of selected genes and the expression of the differentiation marker GFAP. After two or five days of differentiation we found both NHNP cells with weak GFAP staining close to the nucleus and NHNP cells with a strong GFAP staining throughout the cytoplasm of the cell body and the appendages. This staining pattern was reproducible in several biological replicates and indicates different stages of the differentiation process at the two time points.

**Figure 4:**
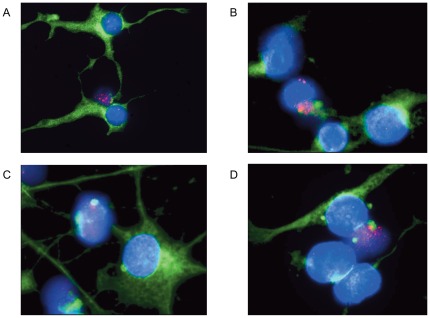
FISH and immunofluorescence analysis. FISH with *GINS2* specific BAC (pink) and simultaneous immunofluorescence staining with GFAP (green) revealed *GINS2* amplification in cells with beginning GFAP expression after 5 d of differentiation (A). FISH with *CDK4* specific BAC (pink) and immunofluorescence staining with GFAP revealed *CDK4* amplification in cells with beginning GFAP expression after 2 d of differentiation (B). FISH with *CDK4* specific BAC (pink) and immunofluorescence staining with Tubulin-ß-III-chain (green) revealed *CDK4* amplification in cells with beginning Tubulin-ß-III-chain expression after 11 d of differentiation (C and D). Nuclei were counterstained with DAPI. Size calibration bar = 5 µm.

While cells with strong GFAP expression showed a normal copy number for all genes tested, e.g. *CDK4* and *GINS2*, cells with weak GFAP expression showed amplifications of *CDK4* and *GINS2* after 2 d and 5 d (Figures 4 A, B). While cells with a weak GFAP expression were localized in or near the sphere, cells that had migrated out of the sphere revealed a stronger expression of the differentiation marker GFAP and a differentiated morphology of the cell body with typical appendages. Similar results were obtained with immunofluorescence staining using the differentiation marker Tubulin beta-3 chain. Notably, after 11 days of differentiation we still found *CDK4* amplification in NHNP cells with weak Tubulin beta-3 chain expression (Figure 4 C, D).

## Discussion

SFME cells are non-tumorigenic and display characteristics of progenitor cells of the central nervous system [10]. Addition of FCS up-regulates GFAP expression indicating the capacity to differentiate into astrocytes [10]. The SFME cell line had undergone 96 population doublings in serum free medium and revealed double minutes in one percent of the cells [6]. A higher percentage of double minutes were detectable in SFME cells that were capable of growing in FCS containing medium. This increase in percentage of cells with double minutes under differentiation promoting conditions prompted us to investigate gene amplification during differentiation.

Here we decided to use human neural progenitor cells that were grown as spheres and that were capable of differentiating into neurons and astrocytes. There is early evidence that cells with glial morphology migrate out of the spheres as result of an induced differentiation [11]. A more recent study reported human NPCs (neural progenitor cells) that expressed both GFAP and Tubulin beta-3 chain in the migration area 24 h after differentiation induction [12]. NHNP sphere cells used in this study revealed cells with glial morphology that migrated out of the sphere a short time after differentiation induction. We were able to confirm GFAP and Tubulin beta-3 chain expression in the migration area 24 h after differentiation induction. In our study gene amplifications appear to occur preferentially in cells that still localize in or close to the sphere. From gene amplification analysis in glioblastoma cells it is known that amplifications can be lost [13]. In this study we can raise the hypothesis that cells with amplifications die upon differentiation or that differentiated cells have lost their amplifications. Further investigations will be necessary to determine whether neural progenitor cells show amplification as a prerequisite for differentiation or whether the differentiation process is the prerequisite for amplification.

Since small focal gains likely represent copy number variations that are commonly found in the human genome, we considered only loci larger than 250 kb. Our amplification analysis after two and five days of *in vitro* differentiation revealed a complex genome-wide amplification pattern with 66 or 93 amplified loci. The size of the amplified chromosome regions was between 250 kb and more than 10 Mb. Array-CGH data were carefully interpreted. We are aware that a gain in log_2_ ratio value of 0.25 can be regarded as gain of one copy. But these calculations were only true when analyzing a homogenous cell population. Here we analyzed a very complex cell population with many cells in differing stages of differentiation. Even after 7 d or 11 d of differentiation we still find cells with amplifications and cells with a weak GFAP or Tubulin ß III staining likely indicating early stages of the differentiation process in these cells. It is very likely that we still missed amplifications in other chromosomal regions by our array-CGH approach because the number of cells with those amplifications is too small or the level of amplification is not high enough for detection by this means. This is further supported by the fact, that in our array-CGH analysis *CDK4* gene amplifications were only detected after 5 d of differentiation. But FISH analysis on NHNP cells differentiated for 2 d already revealed *CDK4* amplifications as shown in Figure 2, 3.

**Figure 5:**
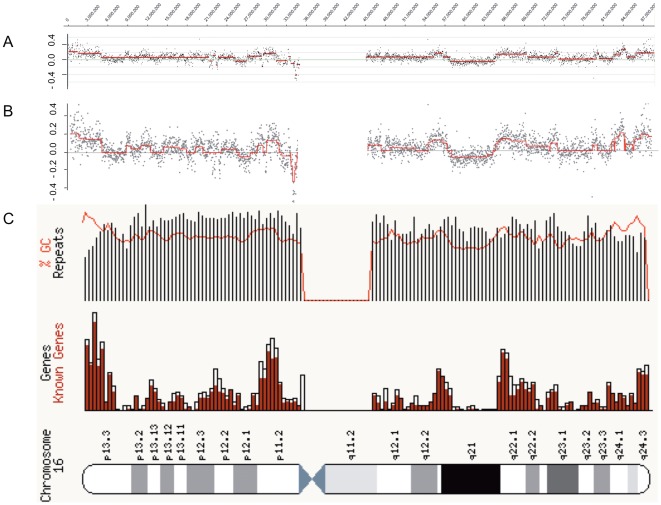
Correlation of gene content to chromosome region. The correlation is shown for human chromosome 16 with several amplified and one deleted region in NHNP cells after 5 d differentiation. The log_2_ ratio profile of the 10× window averaged data is presented at 25 kb resolution (A). Fused lasso analysis is performed using the same data points (B). GC repeats, gene content and banding pattern is shown as indicated by Ensembl genome browser (C). Both the log_2_ ratio profile and the fused lasso analysis indicate an overlap between amplifications and regions with high GC and gene content and an overlap between deletion and regions with low GC and gene content.

Analysis of these chromosome regions revealed hundreds of genes that were involved in this amplification process. Besides amplifications, we also detected deletions that were mainly localized in chromosome regions lacking genes or contain only few genes. In contrast, amplified chromosome regions mainly map within chromosome regions with a high gene density (Figure 5).

As stated above, gene amplification is a hallmark of many human tumors including brain tumors. Several chromosomal regions amplified in neural progenitor cells contain genes that are also amplified in glioblastoma including *CDK4*, *SOX13*, *TERT*, *ABCC3*, *RANBP1*, *MDM4*, *CAMK2A*, *ID2* and *FUT1* [14]–[18]. In addition, we found an overlap between chromosomal regions amplified in NHNP cells that were differentiated for 2 days and 5 days, and gained chromosome regions in 251 glioblastoma deposited in the TCGA data collection [19].

Gene amplifications in normal human cells as physiological process have not been reported yet. Since our *in vitro* study was focused on a narrow time window of few days during the differentiation process and since amplifications were found only in a smaller number of cells, we would not expect to see readily identifiable amplification events in human normal tissue. Further light onto the amplification process will be shed by comparative analyses of this process in other mammals to see if and to what extent amplification can be found during cell differentiation in other species.

## Materials and Methods

### Cell culture and differentiation

NHNP cells were obtained from Lonza (Verviers, Belgium) as cryopreserved sphere culture and were cultivated in maintenance medium (NPMM) containing EGF and bFGF for 24 h after thawing. NHNP cells were primary cells in P1 established from human embryonic brain (17WG, male and 18WG, sex unknown). Spheres were not expanded or cultured after these initial 24 h. Spheres were seeded on laminin-coated glass slides (autoclaved) and allowed to attach to the surface for 5 minutes. Spheres were differentiated using differentiation medium (NPDM) supplemented with BDNF (25 ng/ml).

### DNA preparation

Genomic DNA was extracted from cell cultures and normal blood lymphocytes using NaCl/chloroform extraction. Control genomic DNA was mixed from male and female healthy blood lymphocytes to minimize for normal CNV detection.

### Array preparation, hybridization and detection

NimbleGen 3x720K whole genome array hybridization was done using the certified full service of NimbleGen 3x720K human whole genome array hybridization from ImaGenes Berlin, Germany. Detailed information on data analysis is described at http://www.nimblegen.com/products/cgh/wgt/human/3x720k/index.html. Array-CGH analysis was performed on primary NHNP cells at time point zero, and after 1 d, 2 d and 5 d of differentiation. The array-CGH experiments were done with independently derived primary cells. Array data were deposited in GEO (GSE30636).

### Fluorescence in Situ Hybridization and Immunofluorescence staining

BAC clones were from RP-11 (http://www.chori.org/bacpac/) libraries of the Welcome Trust Sanger Institute and cosmid clone for *CYP27B1* (LLNLc132M0263Q2) available from ImaGenes GmbH, Germany.

BAC probes were directly labeled using High Prime Labeling System (Roche Molecular Biochemicals, Germany). 1 µg of BAC-DNA each were labeled with Cyanine-3-dCTP (Cy3) or Cyanine-5-dCTP (Cy5) (PerkinElmer, Germany), according to the manufacturers instructions. 60 ng of Cy3-labeled and/or Cy5-labeled probe DNA were precipitated in the presence of human Cot-1 DNA. Samples were resuspended in hybridization mix (50% formamide, 2×SSPE, 10% dextrane sulphate and 4% SDS).

NHNP sphere cells were grown on laminin-coated slides under differentiation promoting conditions. Slides were washed once with PBS and methanol fixed 10 min at −20°C. Slides were treated for 5 min in 0.02% Tween-20/PBS.

For FISH with simultaneous immunofluorescence staining, slides were RNase treated (100 µg/ml RNaseA in 2× SSC) for 15 minutes at 37°C. Postfixation was done by 1% formaldehyde/1× PBS for 10 minutes at room temperature. Slides were blocked with goat serum and incubated for 1 h with antibodies either chicken polyclonal to GFAP (ab4674, Abcam) or rabbit polyclonal to neuron specific beta-III-Tubulin (ab18207, Abcam) and detected using Alexa-488 coupled secondary antibodies. Finally, slides were dehydrated by an ascending ethanol series (70%/80%/96%) and air-dried.

For FISH without simultaneous immunofluorescence staining, slides were RNase treated (100 µg/ml RNaseA in 2× SSC) for 30 minutes at 37°C and pepsin treated (0.005% in 0.01 M HCl at 37°C) for 10 minutes. Postfixation and dehydration was done as described.

Both immune fluorescence analyses with and without FISH were done in biological replicates with primary cell cultures that were independently derived from human embryonic brain.

### Hybridization

Labeled BAC probes were applied to the slides and denatured for 2 min. at 80°C. Hybridization was done in a humid chamber at 37°C for 16 h. Post hybridization washes were performed in 50% formamide/2× SSPE (4×5 minutes; 45°C) followed by 0.1× SSPE (3×5 minutes) at 60°C. Nuclei were counterstained with DAPI (4′,6′-Diamidino-2-phenylindole) (1 µg/ml in PBS) for 4 minutes and mounted with VectaShield mounting medium (Vector Laboratories, Orton Southgate, England) for microscopic analysis.
